# Applications of Machine Learning in Alloy Catalysts: Rational Selection and Future Development of Descriptors

**DOI:** 10.1002/advs.202106043

**Published:** 2022-03-01

**Authors:** Ze Yang, Wang Gao

**Affiliations:** ^1^ School of Materials Science and Engineering Jilin University Changchun 130022 P. R. China

**Keywords:** alloys, heterogeneous catalysis, machine learning, reactivity descriptors, structure‐property relationship

## Abstract

At present, alloys have broad application prospects in heterogeneous catalysis, due to their various catalytic active sites produced by their vast element combinations and complex geometric structures. However, it is the diverse variables of alloys that lead to the difficulty in understanding the structure‐property relationship for conventional experimental and theoretical methods. Fortunately, machine learning methods are helpful to address the issue. Machine learning can not only deal with a large number of data rapidly, but also help establish the physical picture of reactions in multidimensional heterogeneous catalysis. The key challenge in machine learning is the exploration of suitable general descriptors to accurately describe various types of alloy catalysts, which help reasonably design catalysts and efficiently screen candidates. In this review, several kinds of machine learning methods commonly used in the design of alloy catalysts is introduced, and the applications of various reactivity descriptors corresponding to different alloy systems is summarized. Importantly, this work clarifies the existing understanding of physical picture of heterogeneous catalysis, and emphasize the significance of rational selection of universal descriptors. Finally, the development of heterogeneous catalytic descriptors for machine learning are presented.

## Graphical abstract

This review briefly retrospects the applications of machine learning methods in various alloy catalyst systems, and summarizes several representative categories of descriptors for various alloy catalysts. Based on the machine learning scheme, not only the existing understanding of the physical picture in the study of heterogeneous catalysis is clarified, but also the significance of rational selection of descriptors is emphasized.

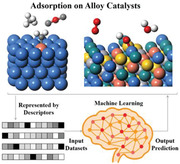

## 1 Introduction

**Figure 1:**
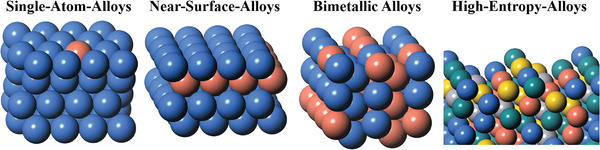
The increasing complexity of structure and composition of various alloy catalyst models.

Alloy catalysts have shown extraordinary catalytic ability in a variety of catalytic reactions of interest, including carbon dioxide reduction reaction (CO_2_RR), ^[^ ^1^ ^]^ hydrogen evolution reaction (HER),^[^ ^2^ ^]^ oxygen reduction reaction (ORR),^[^ ^3^ ^]^ nitrogen reduction reaction (NRR), ^[^ ^4^ ^]^ and ammonia decomposition reaction,^[^ ^5^ ^]^ etc. It is well known that alloying combines together the intrinsic properties of different elements to obtain a unique catalytic performance.^[^ ^6^ ^]^ For the design of alloy catalysts, the main challenge is to fine‐tune their activity, selectivity and stability simultaneously.^[^ ^7^ ^]^ Depending on the structure and composition, alloys exhibit increasingly complicated properties, from single‐atom‐alloys (SAAs), near‐surface alloys (NSAs), bimetallic alloys to high‐entropy‐alloys (HEAs), as shown in **Figure** **1**. To obtain unusual catalytic activity and selectivity, the local active centers are constructed by dispersing the catalytic active atoms on a host surface for SAA systems.^[^ ^8^ ^]^ For NSA systems, the near‐surface chemical properties are tuned by introducing the secondary alloy solutes.^[^ ^9^ ^]^ The unique point‐defect structures usually enable SAAs to break the scaling relationship of conventional transition metal (TM) catalysts, while NSAs have been a long‐term and in‐depth topic in the field of heterogeneous catalysis. Bimetallic alloys can be complex and have been intensively studied, due to the diverse element combinations and variation of proportion, as well as the order, strain, charge and other factors. The further study of alloying effect promotes the development from binary and ternary alloys to the multicomponent HEA systems.^[^ ^10^ ^]^ Owing to the inherent surface complexity, the influencing factors of binary alloys will become more complex in HEAs. HEAs can regulate the electronic structures to a great extent by randomly mixing multiple elements, and thus serve as a platform for the construction of potential catalysts with multiple active sites.^[^ ^11^ ^]^ For catalysis, bimetallic alloys have a more solid research foundation, while the researches of HEAs are still in the embryonic stage. Apart from the contribution of electronic structures of alloys to catalysis, the surface geometry of alloys also greatly affects the catalytic performance due to the different crystal facets and defects.^[^ ^12^ ^]^ For example, the alloy nanoparticles (NPs) with atomic‐scale defects, are of great significance for the quantitative modulation of geometric effects on catalytic activity.^[^ ^13^ ^]^ However, the exploration and discovery of potential alloy catalysts are still in infancy stage, due to the complex geometry and alloying effect of alloy catalysts and the limit of time‐consuming conventional experimental/theoretical methods.^[^ ^1b^ ^]^

For a given catalytic reaction, there are always multiple reaction pathways and competitive reactions simultaneously. The process of heterogeneous catalysis is usually complicated by the presence of multiple intermediates, which determine the reaction thermodynamics and kinetics.^[^ ^14^ ^]^ Moreover, the coupling of the various pathways makes it more difficult to understand the entire reaction mechanism. For instance, the reaction mechanism of CO_2_RR is still a controversial issue, mainly due to the competitive reaction pathways corresponding to the diverse products of CO_2_RR, as well as the inevitable side reaction of HER. Specifically, the key selectivity‐determining steps of O—H, C—H, and C—C bond formation, which lead to various hydrocarbon products, are still intensely debated.^[^ ^15^ ^]^ ORR is also a challenging catalytic reaction, because catalysts must be stable under extreme corrosion conditions and exhibit the moderate activity to activate O_2_ and to release H_2_O.^[^ ^16^ ^]^ For catalysis on complex alloys, the high‐throughput calculations and large‐scale screening by density functional theory (DFT) methods have always been a kind of high‐cost solutions. Therefore, it is definitely necessary to take advantage of the predictive power of machine learning (ML) techniques, which can capture the structure‐property relationship with a much lower cost.^[^ ^17^ ^]^ ML can not only deal with a large number of data rapidly, but also help establish the physical picture of heterogeneous catalysis.^[^ ^18^ ^]^ With the enrichment of material databases and the enhancement of computing power, ML techniques have been widely used in the prediction of the activity, selectivity and stability of catalysts.^[^ ^19^ ^]^ According to the well‐known Sabatier principle, moderate binding strength of key intermediates should be carefully tuned to achieve the highest activity.^[^ ^20^ ^]^ The adsorption energy of key intermediates is thus usually used as the numerical target variable in the ML scheme of heterogeneous catalysis.^[^ ^21^ ^]^ However, the inherent problem in the large‐scale screening of catalysts is that expensive DFT calculations are still required to obtain the adsorption energy of each intermediate of interest. Therefore, it is essential to identify the intrinsic properties of catalysis, i.e., fingerprints or descriptors, which are not only closely related to the adsorption properties, but also effectively obtainable through the theoretical methods, preferably those properties easily accessible. The appropriate descriptors that reflect prior knowledge of application domain, are also one of the most important ingredients for ML. To date, generous efforts have been spent in the investigation of descriptors of catalysts.^[^ ^22^ ^]^ More specifically, the basic properties of active elements in catalysts, and the electronic structure and local geometry of substrate surfaces have been investigated and utilized as the descriptors of catalysts in ML schemes.

In this review, we attempt to provide an overview of some recent successful applications of ML techniques in alloy catalysts, and discuss various reactivity descriptors in different alloy catalyst systems. After a brief outline of basic concepts and workflow of ML scheme, we will introduce the development of ML in the design of alloy catalysts and focus on the opportunities and prospects of catalysis descriptors in this field.

## 2 Machine Learning Concepts

Taking into account both efficiency and cost, ML has been gradually applied in numerous fields of material science, such as material discovery, structure analysis, property prediction, reverse design and so on. Suitable for solving large‐scale combinatorial space, nonlinear process and other complex problems, ML can effectively save the material resources and shorten the research and development cycle. In the application of ML, the most influential factors are the appropriate algorithm and effective descriptors. ML algorithm realizes the human‐like learning and prediction of machine for different situations through a variety of mathematical logics. Appropriate descriptors determine the upper bound of prediction accuracy of ML algorithm. Besides, there are other important factors in the ML process that also affect the ultimate performance of ML models. The quantity and quality of original data in databases are the basis of ML training, while the model validation is helpful for the rational selection of models, datasets and their representations.

### 2.1 Workflow of Machine Learning

**Figure 2:**
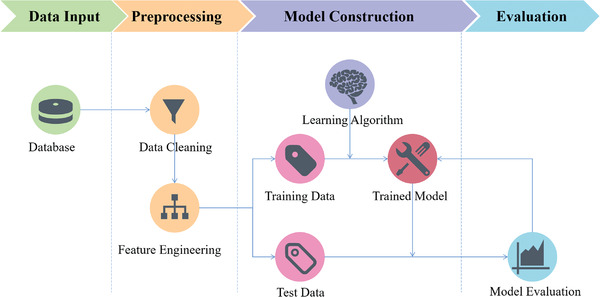
Schematic diagram of machine learning workflow.

Generally speaking, the workflow of ML is to build models based on the existing data and the selected algorithm, to optimize the models continuously, and to predict the target eventually, as illustrated in **Figure** **2**. The construction of standardized data set demands preprocessing, i.e., data cleaning, which reviews and verifies data to remove duplicate information, correct existing errors and provide data consistency.^[^ ^23^ ^]^ Feature engineering mainly deals with the feature extraction and dimension processing of data set, and it is often considered as the most creative part of data science.^[^ ^24^ ^]^ After splitting the training set and test set, an algorithm can learn in a certain way according to the provided data. To avoid over‐fitting and evaluate the generalization ability of a model, it is common to use about 20% of available data as the test set when performing a supervised ML process, in which the test data shall not be used for model adjustment and optimization. Then, the generalizability of a model is verified by the model evaluation on the test set and the hyperparameter settings are adjusted to further optimize the model. With the increase of training times, the model can continuously learn and improve its performance progressively.

### 2.2 Material Databases

The quantity and quality of original data in databases are the basis of ML training. The data collection methods should minimize the noise and ensure the unbiased sampling. For heterogeneous catalysis, the related data are usually collected through experiments and ab initio calculations. However, the results cannot be naively unified on an equal footing due to the systematic errors caused by the different experimental conditions and/or computational parameter settings. Therefore, the key is the construction of the standardized database of various materials. Recently, some popular materials databases for inorganic crystals have been a good choice for ML, including Materials Project,^[^ ^25^ ^]^ Open Quantum Materials Database (OQMD),^[^ ^26^ ^]^ Inorganic Crystal Structure Database (ICSD),^[^ ^27^ ^]^ Cambridge Crystallographic Data Centre (CCDC),^[^ ^28^ ^]^ American Mineralogist Crystal Structure Database (AMCSD),^[^ ^29^ ^]^ etc. Most of these databases provide the open‐source, user‐friendly, and interoperable Application Programming Interfaces (API).^[^ ^30^ ^]^ For heterogeneous catalysis, it is the substrate‐adsorbate interactions that determine the activity and stability of catalysts. CatApp^[^ ^31^ ^]^ and Catalysis‐Hub.org^[^ ^32^ ^]^ have been presented as specialized databases for reaction and activation energies on catalytic surfaces. What's more, Catalysis‐Hub.org contains over 100 000 optimized geometries and adsorption energies obtained from electronic structure calculations. Despite all this, the currently available databases are sometimes still insufficient for the exploration of novel catalysts and the understanding of complex reaction mechanism. Researchers usually have to rely on the high‐throughput calculations to build their own data sets. There are a variety of existing open‐source automated packages for the high‐throughput ab initio simulations, such as Atomic Simulation Environment (ASE),^[^ ^33^ ^]^ Python Materials Genomics (pymatgen),^[^ ^34^ ^]^ Automated Interactive Infrastructure and Database (AiiDA),^[^ ^35^ ^]^ etc. These packages provide modules to perform a variety of simulation tasks, including but not limited to the single‐point energy calculation, geometry optimization, molecular dynamics, and transition‐state search.

### 2.3 Feature Engineering

The features of data determine the upper limit of ML, while the algorithms only make the models as close to the upper limit as possible.^[^ ^19h^ ^]^ Feature engineering is the process of transforming the original data into some training features, and its main goal is to obtain a better sample set to train the learning model. Features should preferably have physical meanings and be accessible, i.e., easy to compute or look up. Ideally, the promising descriptors can directly describe the activity and stability and infer the desired catalysts with a minimum of computation.^[^ ^36^ ^]^ For heterogeneous catalysis, it is crucial to transform the non‐numerical physical or chemical properties of substrates and adsorbates into efficient numerical descriptors that can be recognized by the ML algorithms.^[^ ^19j^ ^]^ Since heterogeneous catalysis is a multi‐scale phenomenon, it is generally described by various information based on the macroscopic, atomic and electronic properties. The synthesis conditions and mesoscale structures are popular for the description of high‐throughput experiments, while most of the computational researches are based on the electronic‐structure methods due to their accuracy in describing the chemical bonds.^[^ ^37^ ^]^ The choice of optimal descriptors, however, usually requires the expert knowledge of learning algorithms as well as the domain scientific problems. To investigate the activity of catalysts at the atomic scale, the direct mapping from the structure and composition of an active phase to its performance, i.e., the structure‐property relationship of catalysts, is particularly significant. Therefore, the interpretability of ML models is usually as important as their prediction accuracy.^[^ ^38^ ^]^ However, the model that can make the most accurate prediction is usually obtained through the more complex feature space and decision rules, which is hardly interpretable and hinders the further understanding in the application domain. There are indeed several ways to get feature “importance”, but there is no strict consensus on the meaning of the word as often.^[^ ^38^, ^39^ ^]^ In scikit‐learn, the implemented importance is defined as the average total reduction of node impurity over all decision trees of ensemble, and is sometimes referred to as gain importance or average impurity reduction.^[^ ^40^ ^]^ Although there are different importance evaluation indexes based on the algorithm principle in different ML algorithms, these indexes are still used as an important reference to evaluate the importance of features to a certain extent. Feature importance analysis is thus helpful for the preliminary screening in feature engineering and the post‐assessment of the correlations between features and ultimate model performance. The selection of features and the construction of descriptors of catalytic materials are the core content of this review, and the specific examples will be introduced in detail in Section 3. The reactivity descriptors developed by predecessors include basic elemental properties, electronic descriptors, geometric descriptors and their derived properties. For the realization of globally universal descriptors, it is crucial to design and develop descriptors that can apply to most catalysts and reactions.

### 2.4 Machine Learning Algorithms

**Figure 3:**
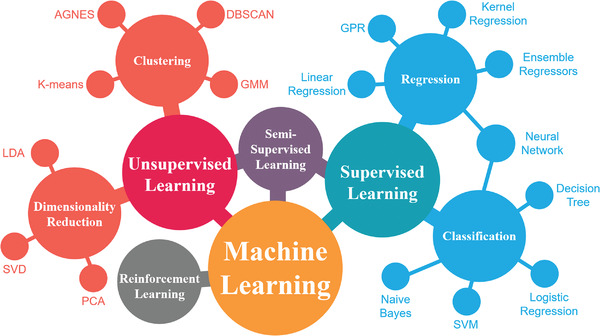
Mainstream machine learning algorithms and their application classification.

At present, ML algorithms can be divided into several categories in principle as shown in **Figure** **3**, including supervised learning, unsupervised learning,^[^ ^41^ ^]^ semi‐supervised learning,^[^ ^42^ ^]^ and reinforcement learning.^[^ ^43^ ^]^ In supervised learning, each sample consists of an input object (usually a vector) and an output value (also known as a label that plays the role of supervision). Supervised learning algorithms analyze the training data and generate an inference function to map on new samples. In unsupervised learning, a machine learns to observe features of various data and explore underlying laws, without demand of the label information. Semi‐supervised learning combines together characteristics of supervised learning and unsupervised learning, mainly considering how to make use of the minor labeled sample set and the major unlabeled sample set for training and prediction. Reinforcement learning, which is famous as AlphaGo,^[^ ^44^ ^]^ obtains learning information and updates model parameters by receiving feedback from the environment without any prior data. So far, most of the ML algorithms used to explore heterogeneous catalysis are supervised learning algorithms. Some existing open‐source packages can help non‐expert users build ML models conveniently, such as scikit‐learn,^[^ ^39a^ ^]^ Tensorflow,^[^ ^45^ ^]^ and Pytorch.^[^ ^46^ ^]^

**Figure 4:**
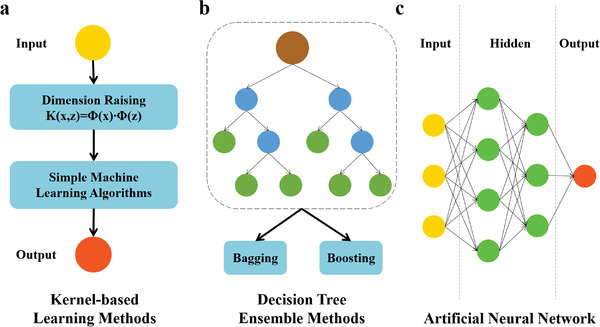
Schematic diagram of algorithmic structures of three categories of commonly used machine learning regression algorithms. a) Kernel‐based learning methods. b) Decision tree ensemble methods. c) Artificial neural network.

The essence of model selection is to select the optimal method and parameters for a specific task. Besides, it is generally accepted that the selection of an optimal algorithm should preferably rely on the known physical picture of descriptors and target properties. First of all, processed data should be analyzed to determine whether datasets have labels, i.e., target variables. Supervised learning algorithms should take precedence when labels exist in datasets, otherwise the situation should be classified as an unsupervised learning issue. Secondly, classification or regression algorithms can be selected according to the type of labels (discrete values or continuous values). Subsequently, one can adopt a variety of algorithms to train data, and then select the optimal one based on the prediction accuracy. In addition, the size of datasets and dimension of features can also play an important role in model selection. As shown in **Figure** **4**, we will briefly introduce the commonly used ML algorithms in heterogeneous catalysis, which mainly fall into following three major categories: kernel‐based learning methods, decision tree ensemble methods, and artificial neural network.

#### 2.4.1 Kernel‐Based Learning Methods

Kernel method has been used in different ML algorithms including kernel ridge regression (KRR),^[^ ^47^ ^]^ support vector regression (SVR),^[^ ^48^ ^]^ Gaussian process regression (GPR),^[^ ^49^ ^]^ etc., which have been widely used in the heterogeneous catalysis. The function of kernel methods is to transform a nonlinear problem in low dimensional space into a linear problem in high dimensional space.^[^ ^50^ ^]^ Specifically, KRR generalizes a linear ridge regression model to a nonlinear model through a kernel method. For a common nonlinear transformation, a large number of inner products in high‐dimensional feature space need to be calculated, while the so‐called kernel trick can solve this issue. Kernel trick calculates features in low dimension in advance and directly presents products in high dimension. Kernel trick can be completed in a single operation, and leave the mapping function and feature space completely implicit. This operation circumvents complex calculations in high dimension and accelerates the procedure of the algorithm. Specifically, kernel functions used to implement the kernel trick include linear kernel function, polynomial kernel function, Gaussian kernel function, sigmoid kernel function, etc.^[^ ^51^ ^]^ Among them, Gaussian kernel function is the most commonly used kernel function and is also known as radial basis function (RBF), which can map data to infinite dimension. With its simple algorithm principle, KRR has recently been applied to the field of face recognition and quantum chemistry.^[^ ^52^ ^]^ Different from KRR, the decision boundary of SVR is the maximum margin hyperplane for the learning sample solution. The few data points nearest to the hyperplane are called support vectors, which play the most important role in SVR.^[^ ^53^ ^]^ Combined with a kernel method for nonlinear regression, SVR is one of the most common kernel‐based learning methods with sparsity and robustness, and has been applied in pattern recognition such as portrait recognition and text classification.^[^ ^54^ ^]^ For GPR algorithm, the difference from other regression algorithms is that the correlation among the features are mainly analyzed, which is reflected by the covariance matrix. Kernel trick is thus used in GPR to reduce the huge amount of calculations of covariance between various dimensional features. Based on the convenience of Gaussian process and its kernel function, GPR has been applied in the fields of time series analysis, image processing and automatic control.^[^ ^55^ ^]^ In a word, in the procedure of kernel‐based learning methods, kernel trick achieves the efficient dimensionality promotion of features, and then makes the fast simple linear algorithm be able to solve the nonlinear problems. However, due to the sensitivity of kernel‐based learning methods to hyperparameters, it is necessary to constantly adjust hyperparameters through cross validation, which will lead to the increase of training cost.

#### 2.4.2 Decision Tree Ensemble Methods

Decision tree is a simple graphic method to analyze data intuitively by applying probability analysis.^[^ ^56^ ^]^ The construction process of decision tree is to select attributes as split nodes and determine the topological tree structure. Owing to the simple structure and strong interpretability, decision tree requires neither much domain knowledge from users, nor the data standardization or hyperparameter adjustment. However, the simple structure of decision tree also suffers instability and the tendency of overfitting. The idea of ensemble methods is to combine together several weak learning models to produce a new strong learning model.^[^ ^57^ ^]^ Therefore, the combinations of decision tree algorithm and ensemble methods, including random forest (RF),^[^ ^58^ ^]^ gradient boosting decision tree (GBDT),^[^ ^59^ ^]^ extra tree (ET),^[^ ^60^ ^]^ etc., are more robust for the rapid prediction of large data sources. Specifically, RF is composed of numerous randomly constructed decision trees.^[^ ^61^ ^]^ The ultimate result of RF is determined by voting results of multiple decision trees. Due to the randomness of the bootstrap sampling method and the features selection, RF can be far more robust than a single decision tree, and has been applied in remote sensing and bioinformatics.^[^ ^62^ ^]^ Unlike bagging concept used in RF to generate decision trees in parallel, boosting concept used in GBDT is to serially build the model by considering the results of every previous tree.^[^ ^63^ ^]^ The basic principle of GBDT is to train the weak prediction model according to the negative gradient information of a loss function at each step, and then to combine together the trained weak models in the form of weighted accumulation. GBDT is an accurate and effective off‐the‐shelf procedure that can be used for both regression and classification problems in a variety of areas including Web search ranking and signal processing.^[^ ^64^ ^]^ Moreover, decision tree‐based methods can quantify the importance of a specific input feature for the model accuracy by analyzing the reduction of the error at each node. Because of the simple algorithm principle of decision tree ensemble methods, the training cost of hyperparameters adjustment can be greatly saved, but the generalization ability is relatively poor.

#### 2.4.3 Artificial Neural Network

Artificial neural network (ANN) has become one of the most popular ML algorithms in various fields of materials science due to its accuracy, robustness and flexibility.^[^ ^19^, ^65^ ^]^ Classical applications of ANN involve various fields: pattern recognition,^[^ ^66^ ^]^ signal processing,^[^ ^67^ ^]^ knowledge engineering,^[^ ^68^ ^]^ expert system,^[^ ^69^ ^]^ robot control,^[^ ^70^ ^]^ etc. With the development of ANN algorithms, a large number of ANN variants have been recently used in different practical applications due to their characteristics, such as back‐propagation neural network (BPNN),^[^ ^71^ ^]^ general regression neural network (GRNN),^[^ ^72^ ^]^ extreme learning machine (ELM),^[^ ^73^ ^]^ deep neural network (DNN),^[^ ^74^ ^]^ convolutional neural network (CNN),^[^ ^75^ ^]^ recurrent neural network (RNN),^[^ ^76^ ^]^ generative adversarial network (GAN)^[^ ^77^ ^]^ and so on. Among the recent popular ANN variants, CNN, which introduce the idea of local perception, is suitable for image recognition and speech recognition,^[^ ^78^ ^]^ while RNN, which introduce the idea of time series, is important for natural language processing, speech recognition, handwriting recognition applications.^[^ ^79^ ^]^ GAN is a new framework for generating models through confrontation process in recent years for unsupervised learning on complex distribution, and is usually used for image generation.^[^ ^80^ ^]^ For all kinds of networks, there are always three basic components. The first component is the network architecture, which describes the hierarchy and interconnection of neurons. Each neuron implements a basic computation, typically including a linear weighting of the connection signal and a nonlinear activation function processing. The second component is the activation function which provides the nonlinear fitting ability. The third component is the learning and iterative methods, which search for the optimal value of weight parameters in the network. The outstanding performance of ANN mainly comes from its easy‐training, adaptive structure and adjustable training parameters. In addition to the most commonly used traditional ANN, another neural network (NN) variant, CNN, has recently been used in material science.^[^ ^81^ ^]^ CNN is a kind of feedforward NN with the convolution operations and deeper network structure, and it is one of the representative algorithms of deep learning in the image classification, object recognition and other computer vision fields.^[^ ^82^ ^]^ The connection of convolution layers in CNN is called sparse connection, that is, the neurons in convolution layer are only connected with the adjacent neurons rather than all the neurons in the previous layer. The sparse connection of CNN improves the stability and generalizability of the network structure. Meanwhile, the parameter sharing mechanism of the convolutional kernel reduces the total amount of weight parameters, which reduces the calculation cost and is helpful for the fast learning. For most types of current ANN algorithms, however, the convergence speed is relatively slower than most of the other ML algorithms. What's more, the construction of network topology has great arbitrariness and flexibility, and thus the lack of theoretical guidance leads to poor interpretability and high training cost.

### 2.5 Model Evaluation

Model selection is to select the best algorithm in a certain algorithm class, while model evaluation is to objectively evaluate the prediction ability of the model and to determine the hyperparameter settings. In the practical model evaluation, commonly used evaluation indexes are usually selected to facilitate horizontal comparison among different models, such as the coefficient of determination (R^2^), mean absolute error (MAE) and root mean squared error (RMSE), etc. for regression algorithms.^[^ ^83^ ^]^ For classification algorithms, error rate, accuracy, balanced F score (F1 Score), etc. are usually used to evaluate the model performance.^[^ ^84^ ^]^ Especially for the commonly used binary classifiers, receiver operating characteristic (ROC) curve is one of the most important indexes to evaluate the model performance, and the area under ROC curve (AUROC or AUC) can quantitatively reflect the model performance.^[^ ^85^ ^]^ The purpose of ML is to accurately predict the unknown based on the known, and some random errors can be avoided or reduced by the process of model evaluation. However, a common situation that must be avoided is called over‐fitting, which regards the noise as a general feature.^[^ ^18^ ^]^ In the training process, the issue of over‐fitting can be solved by regularizing the loss function or increasing the size of training set.^[^ ^86^ ^]^ In contrast, under‐fitting means the failure of extracting general features from the training samples, and this issue can be solved by increasing the polynomial dimension and reducing regularization parameters. Cross validation method, a statistical method to evaluate robustness and generalizability, partitions the training sample of size *k* into a calibration sample of size *k*‐1 and a validation sample of size 1 and repeats the process *k* times. The validation set is divided to test the parameters generated by the training set, so as to judge the compliance of these parameters with the data outside the training set relatively objectively and select the optimal model.^[^ ^18^ ^]^ Stability and fidelity of the evaluation results of cross validation method largely depend on the value of *k*, thus the cross validation method is usually called *k*‐fold cross validation, in which the most commonly used value of *k* is 5 and 10. Leave‐one‐out cross validation (LOOCV) method is a special form of cross validation in the case of a small number of data sets, that is, only one sample in the original training set is used as the validation set, and the rest is used as training data. For the case of small data sets, another helpful method is bootstrapping method, which can generate training sets of desired size from the initial data set through the sampling method with replacement.^[^ ^87^ ^]^ However, the distribution of the data set generated by the bootstrapping method differs from that of the initial data set, which will introduce estimation bias. Therefore, when the amount of data is sufficient, cross validation method is more commonly utilized. In addition to the evaluation of prediction performance of model, the efficiency, complexity, robustness and transferability of model should also be considered in the model evaluation.

## 3 Design of Reactivity Descriptors for Machine Learning

Designing effective descriptors is essential for constructing a ML model for catalyst prediction. Diverse alloys, various adsorbates, as well as the complex reaction mechanism caused by their coupling, make it difficult to extract useful descriptors for heterogeneous catalysis. There are several strategies to select the appropriate descriptors of alloy catalysts as input features for a ML model: i) the descriptors should be able to represent electronic and geometric structures of the local environment of surface active sites; ii) the descriptors should reflect characteristics of various adsorbates in heterogeneous catalysis; iii) last but not least, it is necessary to obtain the descriptors directly from databases or by simple DFT calculations as possible, to improve the efficiency of ML schemes.

Most descriptors are based on the known physical picture, including basic elemental properties and other low‐cost computable properties representing electronic and/or geometric structures of catalyst surfaces. In the design of descriptors, researchers often focus on the interactions between descriptors and target variables, and analyze them to further explain and explore the structure‐property relationship of catalysts. In addition, there is another way to construct descriptors by numerical fitting, represented by sure independence screening and sparsifying operator (SISSO) in the framework of the compressed‐sensing based dimension reduction.^[^ ^88^ ^]^ SISSO applies algebraic/functional operators such as addition, multiplication, exponentials, powers, roots, etc. to construct candidate features based on the primary features, and then recognizes the optimal effective descriptors from the immense feature space. Importantly, numerical fitting methods of descriptor construction enable the low‐cost systematic screening of catalysts with high accuracy, which paves the way for active understanding of the uncertainty in catalytic surfaces.

### 3.1 Basic Elemental Property Descriptors

Widely used in ML for predicting alloy performance, basic elemental properties descriptors (BEPD) have the advantages of their accessibility and reliability.^[^ ^81^, ^89^ ^]^ These intrinsic properties, such as atomic number (AN), group number (GN) and period number (PN) in the periodic table, atomic radius (AR), electronegativity (EN), etc., can be obtained directly from the periodic table, handbooks or material databases.

**Figure 5:**
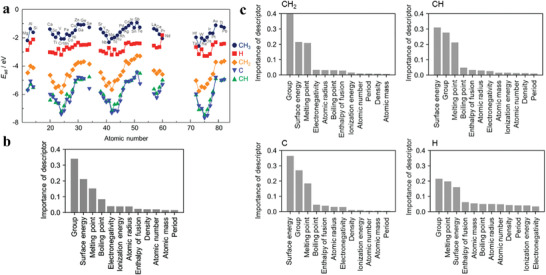
a) DFT‐calculated adsorption energies of CH_3_*, CH_2_*, CH*, C* and H* adsorbates on the Cu‐based SAAs. Feature importance scores of the descriptors for the ETR prediction of the adsorption energies of b) CH_3_* and c) other adsorbates on Cu‐based SAAs. Reproduced with permission.^[^ 89d] Copyright 2018, American Chemical Society.

For SAA systems with a given metal substrate, only the doping element varies from one system to the next, and thus one can use accessible BEPDs of the doping elements to easily describe the differences of SAAs. Toyao et al. employed a simple ML model to predict adsorption energies of the methane (CH_4_) related species on Cu‐based SAAs, based on 12 BEPDs of doped metals, such as GN, PN, AR, etc. (**Figure** **5**).^[^ ^89d^ ^]^ Owing to the large resource of CH_4_ in the form of the natural gas and renewable biogas, there is a strong economic incentive to convert CH_4_ into value‐added products.^[^ ^90^ ^]^ Industrial CH_4_ conversion includes the gas‐based steps to produce synthesis gas. However, catalyst deactivation, caused by coking and sintering of active metals in gas‐based steps, has a negative impact on the overall CH_4_ reforming, but this issue can be overcome by the electrochemical catalysis process.^[^ ^91^ ^]^ A mild electrochemical environment and novel electrochemical catalysts are thus required for the efficient utilization of CH_4_. Among the CH_4_ related species, CH_3_* is the key intermediate for both the reactions of the oxidative coupling of methane to ethylene and the partial oxidation of methane to methanol.^[^ ^92^ ^]^ Therefore, it is crucial to design a surface with strong adsorption of CH_3_* species, to stabilize CH_3_* with respect to CH_2_* and avoid the further dehydrogenation. To design SAA catalysts for the effective CH_4_ utilization, Toyao's work shows a standard workflow of a simple ML scheme. The adsorption‐energy sample set of CH_4_ related adsorbates, including CH_3_*, CH_2_*, CH*, C* and H*, was firstly prepared by DFT calculations. Then the pre‐evaluation for selecting ML algorithms suggests that tree ensemble methods are significantly better than kernel‐based learning methods, and the ETR method can readily predict CH_3_* adsorption energies with the RMSE of 0.24 eV without demanding hyperparameter tuning. Through the feature importance analysis of ETR, it is found that GN, surface energy, and melting point are the top 3 descriptors. When using even only the top 3 descriptors, the robust ML prediction performance remains constant, which also demonstrates the necessity and significance of feature engineering. The investigation of training data ratio shows that 50% of the training data can achieve the moderate accuracy of ETR model, providing guidance for the trade‐off between data availability and prediction performance. The robust model is also applicable for other adsorbates, including CH_2_*, CH*, C* and H*, with the same top 3 descriptors as that of CH_3_*. The model was eventually used to predict the adsorption energy differences (E_CH3_‐E_CH2_) to optimize the efficient utilization of methane. According to their findings, Te, Sn and Mg‐doped Cu‐based SAAs with smaller value of E_CH3_‐E_CH2_ are the most promising candidates, while the elements with larger value of E_CH3_‐E_CH2_ (e.g., Cr, V, and Mo) induce the undesired reactions. Similarly, Lu et al. used the same 5 BEPDs included in Tayao's descriptor set to study the Cu‐based SAAs.^[^ ^89e^ ^]^ Differently, they focused on the active learning of SAA site aggregation energies as well as O* adsorption energies and used the uncertainty quantification ability of GPR algorithm to understand the effect of training dataset selection. The feature importance analysis shows that GN, EN and AR play a more important role, while AN and PN contribute less to the prediction. Interestingly, although the studied adsorbates and algorithms are different in the two references, the importance ranking of the mutual BEPDs for Cu‐based SAAs is surprisingly consistent, that is, from high to low they are GN, EN, AR, AN and PN, respectively. This indicates that the same type of catalyst substrates are likely to have the similar adsorption mechanism for different adsorbates. Additionally, it is worth noting that Dasgupta et al. did just the opposite of the traditional prediction schemes, focusing on the outliers that cannot be identified by the conventional design.^[^ ^89f^ ^]^ Generally speaking, tuning binding energy by alloying (e.g., NSA) is still limited by the Brønsted−Evans−Polanyi (BEP) scaling, i.e., there must be a trade‐off between low activation energy and weak binding.^[^ ^93^ ^]^ As an extreme example of reaction site regulation, SAAs are expected to break the BEP scaling and achieve unexpected performance, due to the extreme dispersion of catalytic metal atoms and the unique synergetic effect with host metal substrates.^[^ ^94^ ^]^ Based on 21 BEPDs, they employed the GPR algorithm to predict reaction energies and adsorption energies for 5 different adsorbates on 300 hypothetical SAAs. Through the local outlier factor analysis, they identified a limited number of SAA outliers, which are most likely to break the established scaling law. Note that the chemistry space of hypothetical SAAs considered in Dasgupta's work involves multiple SAAs with different metal bases. Although eight of the selected BEPDs are the same as those in Toyao's work, their importance ranking is very different from that in Toyao's work. Here one can see that it is not common for BEPD importance analysis to have the similar importance ranking or change trend of different systems. Usually, a consensus of the importance of BEPDs is still hard to reach in various alloy systems. These pioneering works on SAAs have made a good demonstration of ML applications, but it is difficult to extend the ML schemes to more complex alloys, due to the limitation of size and type of the studied systems. Generalization of ML schemes usually demands the introduction of other descriptors to complement the aspects that BEPDs cannot describe, e.g., electronic and geometric characteristics of the local surface environment.

**Figure 6:**
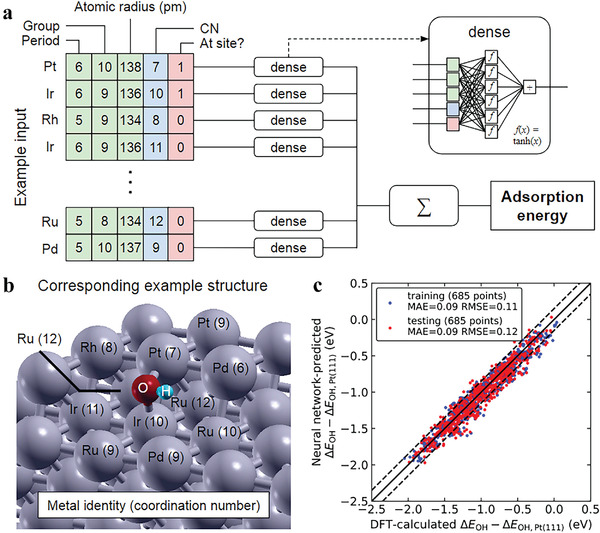
Neural network prediction scheme, example input features, and model parity plot. a) Input features and the example input (green, blue and red blocks indicate ligand, coordination, and nearest‐neighbor descriptors respectively), and the neural network layout depicted in the inset. b) The structure corresponding to the example in panel a, where labeled metal atoms are the active site and its nearest neighbors. c) Parity plot and performance metrics following a random data shuffling and a 50%/50% training‐test data splitting. Dotted lines indicate the errors of <0.15 eV. Reproduced with permission.^[^ ^89h^ ^]^ Copyright 2020, Cell Press.

HEAs are originally known for their novel mechanical properties, and have become a kind of promising catalysts recently, due to their huge chemical space and adjustability.^[^ ^10^, ^95^ ^]^ Two factors that greatly contribute to the variation in active sites are always entangled together, i.e., ligand effect and coordination effect. High‐entropy environment also makes it difficult for BEPDs to predict the catalytic performance. But Lu et al. constructed a clever and simple representation of atomic identity to deal with the difficulty, by introducing the coordination number of metal atoms in active sites as well as the proximity between the adsorbate and the individual metal atom (see **Figure** **6**).^[^ ^89h^ ^]^ They described the atomic identity of quinary IrPdPtRhRu HEAs and captured the influence of ligand effect by using only three BEPDs, including PN, GN and experimentally measured AR. For coordination effect, they adopted the long‐established concept, i.e., coordination number (CN), which is likely the most intuitive geometric descriptor. In addition to the ligand and coordination descriptors, there is another descriptor indicating whether the metal atom is a part of the active site or its nearest neighbor. The ligand, coordination, and proximity descriptors are concatenated into a descriptor vector for each metal atom, and the descriptor vectors from all considered atoms are joined into a matrix, forming a single sample input. To date, ligand and coordination effects have mostly been quantified separately, and their interactions and influence on the catalytic performance still remain unclear, especially for HEAs. To understand the interplay between ligand effect and coordination effect on the catalytic performance of HEAs, Lu et al. utilized the predictive power of NN algorithm to predict adsorption energies of OH* on HEAs under 12 different coordination environments with the MAE of 0.09 eV. Different from the ordinary NN structure, NN prediction of the entire HEA site is achieved by summing individual contributions from each metal atom in the active center corresponding to the input form, which is similar to the approach used in Atom‐Centered Symmetry Functions method.^[^ ^96^ ^]^ Moreover, the model parameters of the network structure are shared for all atoms. This idea of parameter sharing makes the NN model compact (36 parameters in total), and reduces the required training data size. Leveraging the NN design, the contribution of the individual HEA atom to the final OH* adsorption energy can be decomposed by varying the input to the isolated dense layer and observing its output without summation over all atoms. Through the 6‐node dense NN layer, they quantified the exact effects of element identity, CN and proximity. According to the frequency distribution of OH* adsorption energies, the adsorption energy of HEA bridge sites is mainly determined by the mixing contributions of the two active bridge atoms that directly bond with OH*. That is to say, for active centers with different elements, X‐Y, the adsorption energy lies at the average of that of X‐X and Y‐Y. Compared with the central bridge atoms with strong ligand effect, the nearest neighbor atoms have a stronger coordination effect to regulate the HEA catalytic activity. To further simplify the NN model to a linear scaling relationship, they weighted the contribution of each atom to the adsorption energy by its element identity, CN and proximity. In this simplified model, the slight cost of accuracy lost (MAEs of the test set only increase by 0.04 eV) proves the effectiveness of these descriptors. However, DFT modeling of complex, random alloys requires defining a fixed‐size cell, which introduces non‐random periodicity and conflicts with the inherent disorder of HEAs.^[^ ^97^ ^]^ Feugmo et al. have recently proposed a neural evolution structures generation method that combines ANNs and evolutionary algorithms.^[^ ^98^ ^]^ Based on the pair distribution functions and atomic properties, this method can greatly reduce computational cost, making it possible to generate larger cell of HEA structures (over 40 000 atoms) in few hours. The generated HEA structures can be directly used to collect desired properties including structural stability, lattice vibrational property, electronic structure, elasticity, and stacking fault energy. This efficient HEA structure generation scheme can help people understand the complex structure‐property relationship of HEAs more efficiently, and accelerate the exploration and application of novel HEAs. To obtain a better ML model with higher prediction accuracy, researchers often have to select and use multiple BEPDs. Thus, feature sets composed of BEPDs are often redundant, due to the diversity of descriptor types and the intrinsic correlation among them. Though one can analyze the importance of features in an individual ML scheme, a consensus is usually hard to reach in various alloy systems. This may be due to the fact that BEPDs cannot provide sufficient and concise information for the ideal description of complex adsorption mechanism. The essence of this inconsistency is that the real physical picture is still not clear, while BEPDs can only reflect the partial underlying picture. Last but not least, it is well known that the activity and selectivity of heterogeneous catalysis are usually determined by the local environment of active centers on catalysts.^[^ ^19^, ^99^ ^]^ When the local environment of active centers become more complicated, it is unrealistic to accurately predict the catalyst performance from the overall perspective only by the basic individual properties of involved elements.

### 3.2 d‐Band Theory Descriptors

To date, d‐band theory proposed by Nørskov et al. has been proved helpful for understanding the surface chemisorption and heterogeneous catalysis for metallic catalysts.^[^ ^100^ ^]^ In the theoretical framework of tight‐binding approximation, the valence state of a given adsorbate firstly interacts with the metal *s*‐state to produce a broad renormalized state, and then the renormalized state couples with the narrow *d*‐state, which often gives rise to the state splitting, i.e., the bonding and antibonding states.^[^ ^101^ ^]^ The essence of d‐band theory is that the variation of adsorption energies from one TM surface to the next depends largely on the d‐band properties of the surface, such as the well‐known d‐band center. The d‐band center refers to the weighted average energy of electronic *d*‐states projected onto a surface metal atom.^[^ ^101a^ ^]^ Generally speaking, the higher the *d*‐state energy is relative to Fermi level, and the stronger the surface‐adsorbate bond is. However, only the 7 late‐TMs, whose d‐band center and width are independent, strictly follow the d‐band theory model.^[^ ^102^ ^]^ In addition, several early‐TMs and a subset of alloys with relatively small perturbations to the host metal, can be detected by the d‐band model with a reasonable accuracy.^[^ ^20c^ ^]^ Actually, many measured activities cannot be explained by the usual trend of the d‐band center, mainly due to the unconsidered diffusion of energy state.^[^ ^103^ ^]^ For these cases, the correlation between the *d*‐band center and the activity can be improved by introducing the *d*‐band width, owing to the different contributions of the *d*‐band center and width to the adsorption energy of the late and early TMs. By introducing the *d*‐band upper‐edge, the *d*‐band model can better identify Ni, Pd and Pt, but the prediction accuracy is still unfortunately inadequate for other TMs. In the application of ML scheme to the catalytic performance prediction of TMs and alloys, other relevant characteristics of the *d*‐states have been also inputted as descriptors, including the filling, skewness, and kurtosis, which are also important factors to tune the reactivity of various metal surfaces.

**Figure 7:**
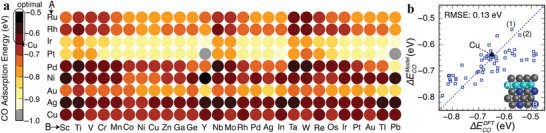
a) Rational screening of CO adsorption energies on the second‐generation core–shell alloy surfaces (Cu_3_B‐A@Cu_ML_) using the developed neural‐network model. b) Parity plot shows a comparison of the CO adsorption energies on selected Cu monolayer alloys calculated using the neural‐network model and self‐consistent DFT. Two alloys, (1) Cu_3_Y‐Ni@Cu_ML_ and (2) Cu_3_Sc‐Ni@Cu_ML_ are identified to have desired CO adsorption energy. The inset in panel b shows the geometric structure of the model system. Reproduced with permission.^[^ ^104^ ^]^ Copyright 2015, American Chemical Society.

As a pioneering work of ML for alloy catalyst design, a chemisorption model based on the ANN algorithm was reported by Ma et al.^[^ ^104^ ^]^ For the CO/CO_2_ electrochemical reduction on Cu(100), the proton‐electron transfer to CO* is the critical step in the C_1_ pathways, while the dimerization of CO* with the sequential proton‐electron transfer governs the onset potentials in the C_2_ pathways.^[^ ^15^, ^105^ ^]^ Thus, they aimed to identify effective descriptors for adsorption energies of the key intermediate CO*, which linearly correlate with those of other intermediates. The strong binding strength of CO* will increase the overpotentials for both the C_1_ and C_2_ pathways, but slightly tends to result in the formation of C_1_ species. The weak bonding strength of CO* leads to a less negative limiting potential for the step of two adjacent CO* to COCOH*, while the thermodynamic driving force from CO* to CHO* varies slowly, thus enhancing the selectivity of C_2_ products. Motivated by the *d*‐band theory, they adopted the characteristics of *d*‐states distribution, including filling, center, width, skewness and kurtosis, together with the delocalized *sp*‐states determined local Pauling electronegativity as primary features. Using a standard feedforward ANN model, they constructed a nonlinear mapping between the descriptor vector and the target CO* binding energy with the RMSE of 0.12 eV. Based on the NN model trained with the bimetallic data set, they identified several promising second‐generation core–shell alloys with the desired CO* binding energy, i.e., 0–0.2 eV weaker than the CO* adsorption on Cu(100) (see **Figure** **7**).^[^ ^106^ ^]^ This type of alloys have been widely studied for many electrochemical reactions, such as ORR, due to the flexibility of reactivity regulation through the strain and ligand engineering.^[^ ^3^, ^107^ ^]^ Statistical analysis of network response to input perturbations further clarifies the underlying factors governing adsorbate‐substrate interactions.^[^ ^108^ ^]^ That is to say, *d*‐band center plays an important role for all the alloys while the shape of *d*‐band has a higher significance for coinage metal alloys. In addition, local Pauling electronegativity determines the substrate‐adsorbate bonding distance and plays an important role in chemical bonding, especially for coinage metal alloys where the *d*‐band is fully occupied and the *sp*‐band interactions dominate. These findings strongly suggest that the inclusion of *d*‐band shape and *sp*‐band properties is crucial for capturing surface reactivity of TM alloys within a broad chemical space. This pioneering study of ML scheme opens up a new way for the further design of complex alloy catalysts. Using Ma's dataset of {100}‐terminated alloys, Noh et al. adopted a non ab initio *d*‐band theory descriptor, i.e., the linear muffin‐tin orbital theory (LMTO)‐based *d*‐band width instead of the DFT calculated *d*‐band width,^[^ ^109^ ^]^ to predict CO* binding energies with less computation cost.^[^ ^110^ ^]^ Combining the active learning algorithm with the non ab initio descriptors, they obtained an active KRR model with the RMSE of only 0.05 eV. Although the LMTO based d‐band width has higher prediction accuracy than the DFT calculated *d*‐band width, the inclusion of the DFT calculated *d*‐band center can still significantly improve the overall accuracy by 0.03 eV (from 0.05 to 0.02 eV). Furthermore, as an example of practical application of the active KRR model, they identified several alloy catalysts for electrochemical reduction of CO_2_, which have an overpotential about 1 V lower than that of Au(100). For the {111}‐terminated bimetallic alloy catalysts, Li et al. presented a holistic NN framework for the rapid screening.^[^ ^111^ ^]^ Based on a thousand of ideal alloy surfaces, they captured the complex adsorbate/substrate interactions in methanol electro‐oxidation. The feature analysis reveals the potential factors for adsorbate‐metal interactions, and provides the physical origin to break the energy scale constraints of CO* and OH*. What's more, a variety of opinions can be drawn from the ML analysis that i) the d‐band features characterized by the lower moments play a more important role than the higher moments; ii) CO* is more dependent on the *d*‐band features than OH*, and iii) the *sp*‐band properties indirectly determined by local electronegativity have a significant effect on OH* than CO*.

In this regard, the *d*‐band theory descriptors benefit from the research basis of the *d*‐band theory itself, and therefore have a high correlation with the activity of catalysts. A large number of studies have shown that the *d*‐band model can well predict the surface reactivity of TMs and some alloys.^[^ ^20^, ^102^, ^112^ ^]^ However, generally speaking, the characteristic parameters of *d*‐band theory, especially the *d*‐band center, demand the time‐consuming DFT calculations for each catalyst material. This disadvantage of *d*‐band theory descriptors limits the transferability and efficiency of their usage in ML schemes.

### 3.3 Local Geometry‐Based Descriptors of Adsorption

The catalytic activity is often determined by several specific surface‐active sites, and the design of active sites is the key to obtain efficient heterogeneous catalysts. Due to the complex electronic properties of alloys, there exist more complex electron transfer between a surface‐active atom and its surrounding local environment. Various coordination environments formed by different facets will also significantly affect the activity of surface sites. On the basis of electronic features, one should also consider geometric features to describe the local environment of catalytic surfaces, which will improve the rationality and comprehensiveness of catalyst fingerprints. Different from the electronic descriptors, the geometric descriptors are mostly used to effectively describe complex NPs with various high index surfaces, whose catalytic behaviors and properties differ from that of bulk metals.^[^ ^113^ ^]^ Moreover, pure metal NPs shed light on the advantages of local geometric descriptors, due to their complex surface structures and high specific surface areas. Generally, ML requires compact input data in the smooth feature space. Thus, a qualified local geometric descriptor needs to fulfill several criteria summarized here, i) it should be invariant to the rotation, translation and homo‐nuclear permutation; ii) it should be uniquely encoded to describe any given structure; iii) it should be continuous in the spanned feature space.

A series of effective local geometric descriptors have been developed and successfully applied to the study of alloy catalysts, such as generalized coordination number (GCN),^[^ ^114^ ^]^ orbital‐wise coordination number,^[^ ^115^ ^]^ Smooth Overlap of Atomic Positions (SOAP),^[^ ^116^ ^]^ Coulomb Matrix (CM),^[^ ^117^ ^]^ Atom‐Centered Symmetry Functions (ACSF),^[^ ^96^ ^]^ Voronoi features,^[^ ^81^, ^118^ ^]^ etc. GCN is a simple extension to the second coordination shell of the well‐known concept of coordination number (CN).^[^ ^113f^ ^]^ The orbital‐wise coordination number quantifies the degree of coordinative saturation of metal atoms and their inclination to form new bonds via the *s* or *d* orbital of an adsorption site. SOAP represents the local environment around a center atom by the Gaussian‐smeared neighbor atom positions with rotationally invariance. CM is a global descriptor based on the pairwise Coulomb repulsion of the nuclei. ACSF uses symmetry function to express the distance and angular interactions between each atom and its neighboring atoms. Voronoi features of atoms in the surface structures provide local environment information in the form of solid angles. Solid angle is the projected area of the shared plane, which is between the adjacent atom and the Voronoi polyhedron of the center atom, on the unit sphere of the adjacent atom. As far as local geometric descriptors are concerned, they are very useful in describing complex geometric structures such as NPs. However, most local geometric descriptors only take into account the structural properties of substrates, without considering their elemental and electronic properties. Thus, even the combination of several effective local geometric descriptors in ML schemes cannot further improve the overall prediction accuracy, due to the redundancy of these input information.

**Figure 8:**
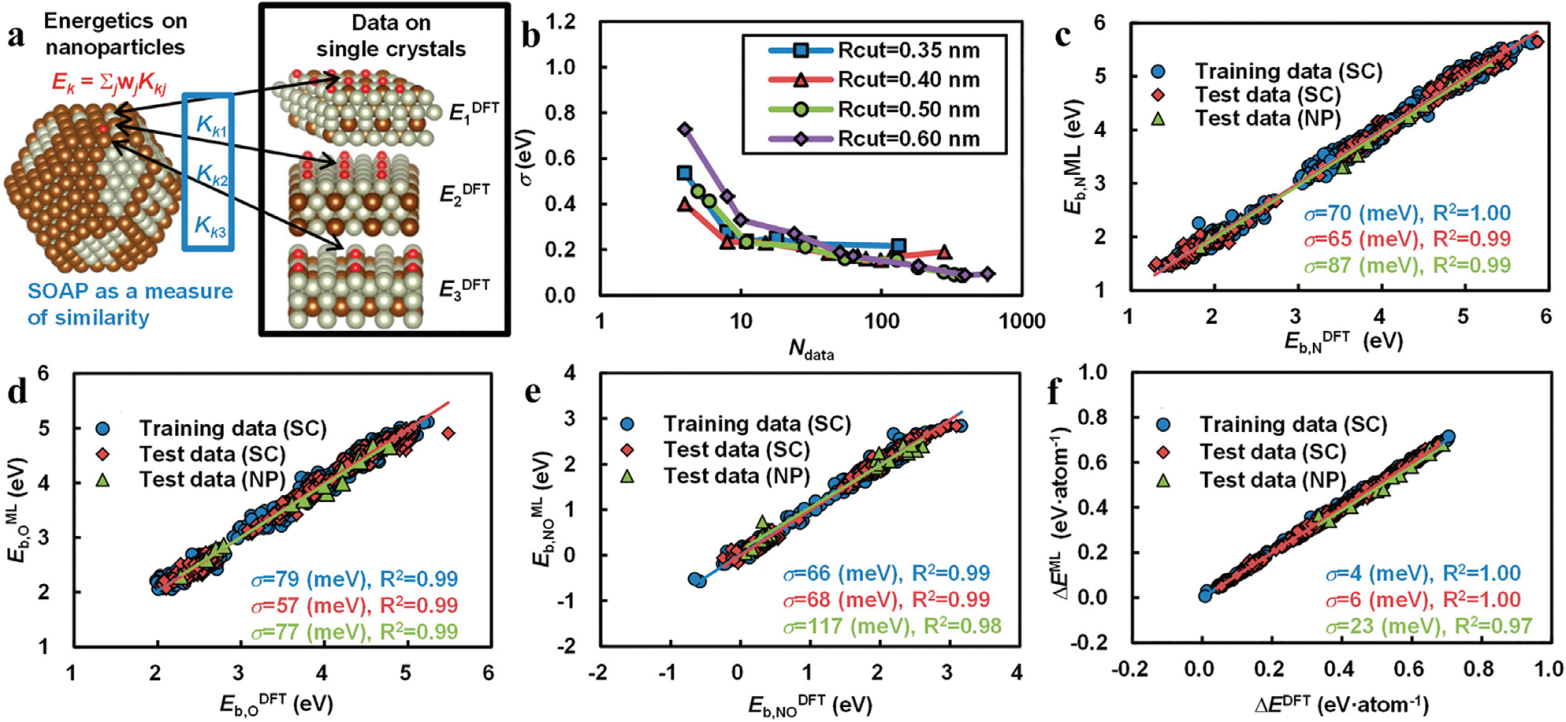
a) Schematic of the algorithm. b) Mean absolute error *σ* in the predicted energies of O* on the Rh_(1‐x)_Au_x_ single crystal surfaces as a function of the number of used training data that equals the number of basis sets in the calculation. c–e) Predicted binding energies of (c) N*, (d) O*, and (e) NO* on Rh_(1‐x)_Au_x_ single crystals and nanoparticles as a function of those obtained by DFT. R^2^ refers to the coefficient of determination. f) Predicted formation energies of Rh_(1‐x)_Au_x_ single crystals and nanoparticles from the pure Rh and Au bulks as a function of those obtained by DFT. Reproduced with permission.^[^ ^113b^ ^]^ Copyright 2017, American Chemical Society.

The element distribution and variation of alloy NPs present new challenges for local geometric descriptors, although some local geometric descriptors can reflect the interactions between atoms of different elements by their distance. Especially in the study of the interactions among multiple elements in alloys, most of the local geometric descriptors are weak and inefficient in describing various alloy systems. Notably, crystal structures of alloy NPs vary with the composition, resulting in the demand of more DFT calculations to capture the surface inhomogeneity and complexity than the ordinary pure metal NPs. Jinnouchi et al. proposed a general ML scheme using SOAP to describe the RhAu alloy NPs with atomic‐scale defects.^[^ ^113b^ ^]^ Catalysis of direct decomposition of nitric oxide (NO) without reductant can meet the increasingly stringent emission requirements of gasoline and diesel engines for small passenger vehicles. Several noble metals (e.g., Rh and Pt) are known to activate the NO decomposition, but the surfaces of these catalysts are usually poisoned by the strongly binding oxygen atoms. It has been shown that alloying the catalysts with Au can improve the desorption rate of oxygen.^[^ ^119^ ^]^ Designing the corresponding alloy NPs can further improve the utilization of noble metal atoms and the catalytic activities of surface sites, because of their high specific surface areas and volume ratios. Relying on the fact that catalytically active sites are determined by their local atomic configurations, Jinnouchi employed a ML scheme and extrapolated the experience to alloy NPs by learning geometrical information of the well‐defined single crystal (SC) surfaces (see **Figure** **8**). The geometrical information of surface sites of both SCs and alloy NPs is evaluated by the local similarity kernel, i.e., SOAP. Local similarity kernel *K_ij_* consists of the overlap integrals between 3D atomic distributions within cutoff radius (*R_cut_*) from the *i*th surface site and those from the *j*th surface site. Correlating catalytic activity with the local atomic configurations, SOAP is applied to predict the activity of direct decomposition of NO on RhAu alloy NPs. The achievable accuracy depends on *R_cut_*, that is, the shorter *R_cut_* leads to the fewer atoms in the range and the smaller dimension of variables composing SOAP. They found that the catalytic activity is volcanically related to the Au atomic ratio (*AR_Au_*) for alloy NPs of any diameter, and the maximal activity and its corresponding *AR_Au_* increase with the decrease in the diameter of NPs. Through the stable atomic distributions determined by Monte Carlo simulations, Au atoms are found to preferentially segregate at the corners and edges of alloy NPs at low *AR_Au_*, and gradually cover the whole surface with the increase of *AR_Au_*. Results of local energetics show that intermediates bind to the active corner sites with moderate binding energy, indicating the mechanism of activity enhancement at the alloyed corner sites. Under the assumption that binding energy is dominated by the local structure, prediction accuracy can be systematically improved by increasing the number of DFT data to cover all the possible local structures of NPs. It should also be noted that this scheme is applicable to any property dominated by the local structure. For HEA surfaces with large numbers of unique binding sites, Batchelor et al. have employed a simple ML model to predict and span out the full set of adsorption energies of OH* and O*. They firstly investigated the stability of IrPdPtRhRu quinary HEAs to form a stable solid solution, based on the atomic radius difference factor, and the ratio taken from the Gibbs free energy of mixing.^[^ ^120^ ^]^ Subsequently, they constructed a simple model to linearly correlate the local environment with the adsorption energies. By counting nearest neighbors, dividing active centers into several zones and considering different alloy elements separately, this model is capable of predicting all possible surface sites. The differences among the selected parameterized zones of the nearest neighbor atoms are attributed to the different interaction strength and coordination numbers for surface and subsurface. Compared with the GCN and orbital‐wise coordination number methodologies, this local geometric representation can better describe the inherent structural disorder on the HEA surface.

### 3.4 Derived Intrinsic Descriptors of Substrates and Adsorbates

Intuitively, the adsorption energy should be a function of electronic and geometric properties of adsorbates and substrates. By correlating intrinsic properties of substrates and adsorbates with the adsorption strength, not only the basic determinants of adsorption energy can be identified, but also the foundation for the rapid estimation of adsorption energy can be established. It is impractical to predict the overall activity of catalyst for a given catalytic reaction only by the basic properties of alloy elements, when the interplay among atoms at the active center of alloys becomes increasingly complex. Similarly, only electronic descriptors or geometric descriptors cannot fully describe the active sites of catalysts either. All the above types of descriptors are only designed for substrates, however, activity and selectivity of the actual catalytic reaction are also strongly dependent on adsorbates. It would be helpful to describe adsorption properties of reactants, intermediates and products on an equal footing for the understanding of the complex mechanism. A comprehensive model should incorporate electronic and geometric effects of substrates and adsorbates together to determine the adsorption, so as to provide an overall physical picture for the adsorption mechanism.

In our previous work, our group has proposed a solution, that is, an entire expression of the adsorption energy based on the electronic descriptor *ψ* and the geometric descriptor $\bar{CN}$ of substrates and the characteristic parameter of adsorbates *α*.^[^ ^121^ ^]^

(1) $$\begin{array}{ccc} E_{ad} & = & 0.1\times \alpha \times \psi +0.2\times \left(1-\alpha \right)\times \bar{CN}+\theta =0.1\times \frac{X_{m}-X}{X_{m}+1}\\ & & \times \;\psi +0.2\times \frac{X+1}{X_{m}+1}\times \bar{CN}+\theta \end{array}$$

(2) $$\psi \;=\frac{{\left(\prod_{i=1}^{N}S_{V_{i}}\right)}^{2/N}}{{\left(\prod_{i=1}^{N}\chi_{i}\right)}^{1/N}}\;$$

where *ψ* is an electronic descriptor of substrates with the form of geometric mean to represent an active center, in which *χ_i_* and S_V_ *_i_* respectively represent the electronegativity and the valence electron number of *i*th atom at the active center. *α* is a characteristic parameter of adsorbates, in which *X_m_* and *X* respectively represent the maximum bondable number and the actual bonding number of the central atom for a given adsorbate. In terms of the *d*‐band theory, the constant *θ* is likely attributed to the coupling between the valence state of adsorbates and the *sp*‐state of metal substrates. Our theoretical framework of the electronic descriptor *ψ* is inspired by the *d*‐band theory proposed by Nørskov et al., which has particularly successfully clarified the adsorption trend of late TMs.^[7, 20b, 101a, 102]^ Combining the *d*‐band theory with the Muffin‐Tin‐Orbital theory, one can obtain that the contribution of *d*‐states to the adsorption strength *E^d^* is proportional to the coupling Hamiltonian matrix element *V_ad_*, which is related to the spatial extent of the metal *d*‐orbital (*r^d^*) and the adsorption distance (*L*), *E^d^* ∝ (*V_ad_*) ∝ (*r^d^*)^3^ */L* ^7^.^[^ ^122^ ^]^ *r^d^* is associated with the *d*‐band center or the number of outer electrons *S_V_*, while *L* can be empirically estimated in terms of the Pauling electronegativity *χ*.^[^ ^3^, ^123^ ^]^ In addition, the electronic descriptor *ψ* reflects the upper edge of *d*‐band and the *p*‐band center of oxides, so as to capture inherent electronic characteristics of adsorption on TMs, NPs, NSAs and oxides.^[^ ^20^, ^123^, ^124^ ^]^ The pre factor of electronic term, $\alpha \;=\frac{X_{m}-X}{X_{m}+1}\;$, can be understood or deduced by the effective medium theory (EMT).^[^ ^125^ ^]^ The pre factor of coordination term, $1-\alpha \;=\frac{X+1}{X_{m}+1}\;$, can be understood from the spirit of bond‐order conservation.^[^ ^126^ ^]^ This intrinsic model incorporates the electronic and geometric effects of substrates and adsorbates together and gives an entire expression of adsorption energy, recognizing the *d*‐band model and the generalized coordination number model. Using the intrinsic model, one can automatically deduce the linear scaling relationship (LSR) and its generalized form, namely the intrinsic correlation between two adsorbate species. This model can also generalize the efficiency of engineering adsorption energy and reaction energy, and naturally deduce the thermodynamic limitations of ORR catalysis. Our descriptors not only provide novel physical insights into the coupling between adsorbates and substrates at the interface, but also offer a theoretical basis for rapid catalyst screening with high accuracy.

**Figure 9:**
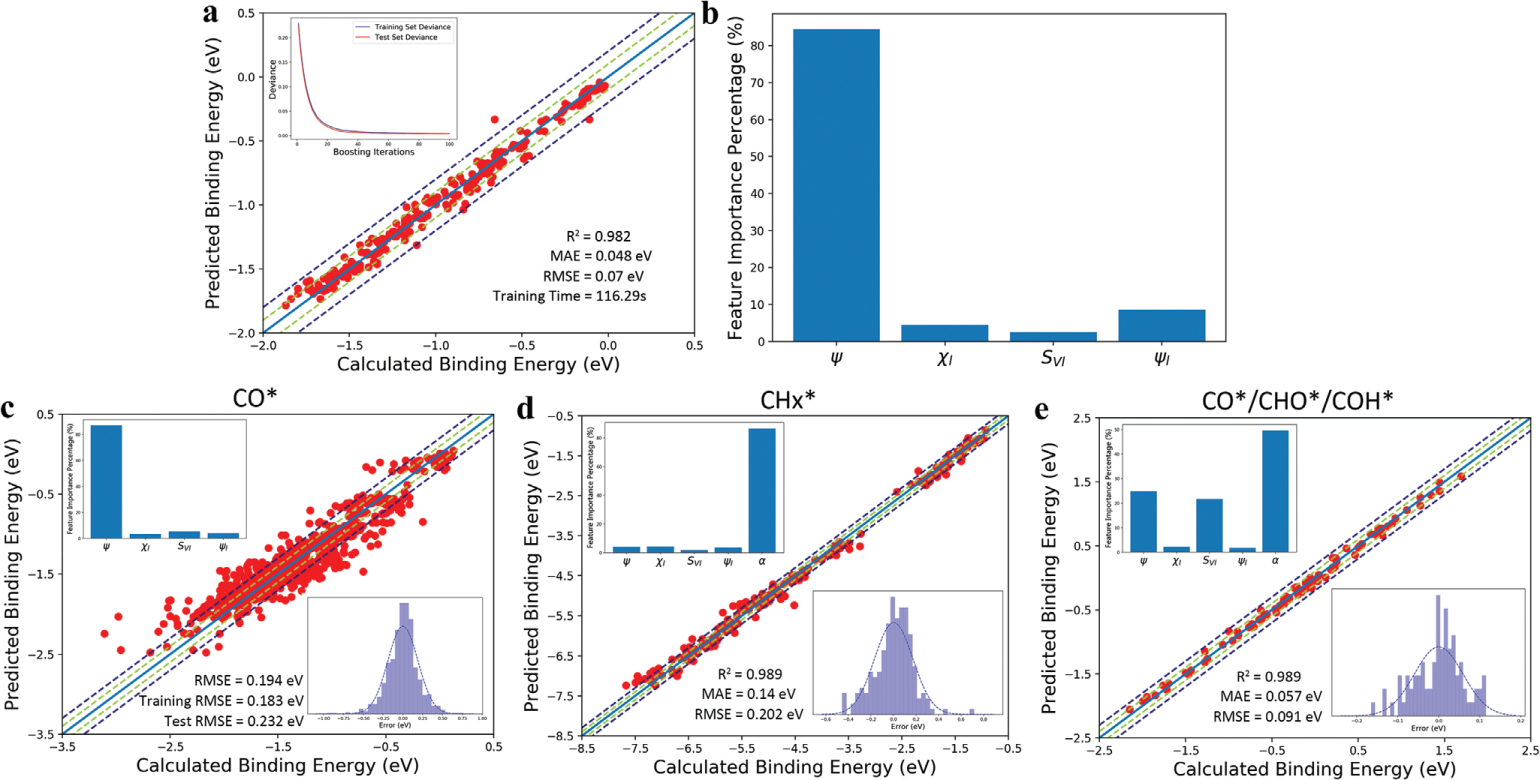
Results of the ML model. a) Fitting results of calculated binding energies and predicted binding energies of CO*. R^2^, MAE and RMSE are computed to estimate prediction errors and total training time for one thousand trials. The inset shows the convergence of GBR model accuracy for five cross‐validation splits of the data. b) Feature‐importance scores of descriptors for the GBR prediction of binding energies of CO* on various alloy surfaces. c–e) Performance of the ML model for predicting CO* binding energies on fcc(111) of NSAs and binding energies of various intermediates on SAAs surfaces. Insets in the lower right corner show the prediction error distribution of the GBR model. Reproduced with permission.^[^ ^127^ ^]^ Copyright 2020, The Royal Society of Chemistry.

In our subsequent work, we adopted the core descriptors in the equation, including the electronic descriptor *ψ* and geometric descriptor $\bar{CN}$ of substrates as well as the characteristic parameter of adsorbates *α*, as input features of our ML scheme.^[^ ^127^ ^]^ Circumventing the usage of expensive quantum‐mechanism calculations, the GBR model accurately predicts the binding energies of various carbon‐terminated intermediates simultaneously and realizes rapid screening in the vast phase space of alloys, as shown in **Figure** **9**. According to the analysis of the reaction mechanism in our ML scheme, we can group the adsorbates by their different coupling mechanisms with substrates. Furthermore, ML results also show that i) the catalytically active centers of metallic materials, especially for various alloys, are highly localized and even down to the single bonding center atom; ii) the adsorption properties of alloys are mainly engineered via modifying the band occupation rather than the electronegativity; iii) the characteristic parameter of adsorbates *α*, which describes reactants, intermediates and products on an equal footing, is helpful to understand the differences of the adsorption mechanisms of various adsorbates and their correlation with the reaction selectivity. In particular, our electronic descriptors for substrates are proved capable of serving as a good alternative to the *d*‐band properties, which further supports the previous finding that the descriptor *ψ* essentially reflects the upper edge of *d*‐states for metallic materials.

**Figure 10:**
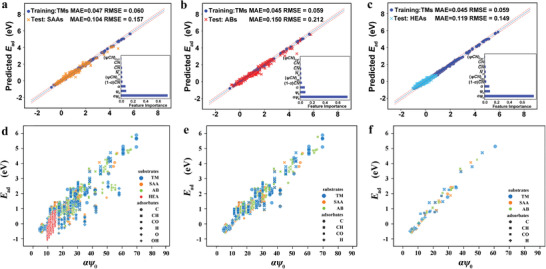
The transferable ML models and the linear relationship between adsorption energies and intrinsic electronic descriptors of substrates and adsorbates. Transferability of ML models in predicting the adsorption energies of a) SAAs, b) ABs and c) HEAs with various adsorption sites, facets and adsorbates by training the data of TMs. Insets show the importance of the corresponding descriptors. The linear relationship between adsorption energies and *αψ_0_* on TMs, SAAs, ABs and HEAs d) with all the considered surfaces and all the adsorbates, e) with all the considered surfaces and the adsorbates C*, CH*, CO* and H*, and f) with the (211) surfaces and the adsorbates C*, CH*, CO* and H*. Reproduced with permission.^[^ ^128^ ^]^ Copyright 2022, The Royal Society of Chemistry.

Based on these descriptors, our group has further proposed a transferable ML model, which not only enables one to estimate adsorption energies of complex alloy systems (AB intermetallics, SAAs and HEAs) only by training the data of pure TMs, but also captures the size‐ and morphology‐effect of adsorption energies on NPs effectively (see **Figure** **10**).^[^ ^128^ ^]^ Specifically, we not only adopted the core descriptors in the equation to describe substrates and adsorbates respectively, but also adopted their coupling terms to directly describe the electronic and geometric effects of the substrate‐adsorbate interactions. This model with nine accessible intrinsic descriptors can capture the subtle variation of adsorption‐energy perturbation caused by the exchange of active‐center atoms on HEAs. By clarifying the correlation between the adsorption energies and the intrinsic electronic and geometric properties of substrates and adsorbates, this model demonstrates that the electronic effects of active centers on adsorption are highly localized even down to the adsorption sites on SAAs, ABs and HEAs. The transferability of our ML model for TMs, various alloys and NPs is attributed to our universal descriptors with clear physical meanings. These descriptors are relatively continuous for various alloys and can thus capture the similarity of different systems. Specifically, *αψ_0_* exhibits a rough linear relation with the adsorption energies on both TMs and alloys with various surfaces, and $\bar{CN}$ also exhibits a rough linear relation with adsorption energies of NPs with different sizes and morphologies. Moreover, this model screens out some potential alloy catalysts for CO_2_RR with reasonable onset potentials. Note that our descriptors have been universally used in theoretical/experimental researches to efficiently describe heterogeneous catalysis on pure TMs, NPs, NSAs, oxides, single atom catalysts (SACs), bimetallic atom catalysts (BACs) and HEAs.^[^ ^121^, ^129^ ^]^ Our descriptor system provides a novel insight into the mechanism of adsorption, and allows rapid screening of potentially interesting systems, all of which provide a guidance for the future material design. Even so, the obtained physical pictures in our descriptor system still await the further generalization to more complex catalyst systems.

### 3.5 Numerical Fitting‐Based Descriptors

The important properties of catalysis are generally determined by several key variables. Commonly, the specific relationship between descriptors and catalytic properties remains unclear. Thus, more accurate and generalizable descriptors may exist but remain undiscovered, and the physical intuition may be limited and unavailable to achieve the systematic improvement. Compared with the physical picture‐based approaches, there is another way to address the issue by constructing descriptors through the numerical fitting methods. By searching for structures and patterns in the data, numerical fitting methods can provide insights for the identification of the structure‐property relationship. Another advantage of data‐driven approaches is that the learning can be systematically improved by enlarging the training data set, while the linear scaling relationship with the rigid format cannot. The key intelligent step of numerical fitting methods is the identification of effective descriptors, which can help ML methods improve model performance more easily.

**Figure 11:**
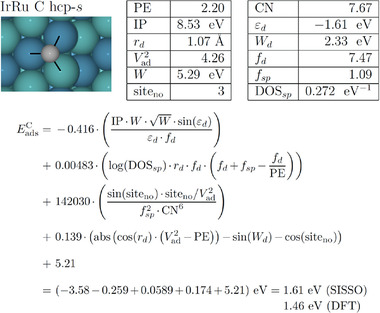
Example of SISSO adsorption energy prediction for C* at an hcp‐s site of the IrRu alloy using the 4D descriptor of Φ_3_ given in an explicit nonlinear functional form owing to the compressed sensing methodology. The tabulated primary features are calculated as averages over the three metal atoms (two Ir atoms and one Ru atom) making up the IrRu hcp‐s site (marked with black lines). The shown fitting coefficients are specific for C*. For ease of reading, their units are not shown; these depend on the units of the primary features entering each feature to ensure that the adsorption energy comes out in eV. Reproduced with permission.^[^ ^22j^ ^]^ Copyright 2019, American Chemical Society.

Compressed sensing (CS), a developed technology in the field of signal processing, provides a simple, general and effective method to find the key description variables of materials.^[^ ^130^ ^]^ The main idea of CS is to accurately recover the sparse high‐quality signal, requiring only a very small observation set. Another practical important feature of CS is that it can tolerate the noise in the input data and to deal with the approximately sparse signals. Rather than attempting to develop the physical intuition for the most relevant variables, CS framework allows the inclusion of essentially all possible basic functions to directly establish physical models. Moreover, CS, which is a real paradigm shift from the traditional techniques, can identify relevant parameters for any sparse basis‐expansion problem in physics, chemistry and material science. In the framework of CS, Ghiringhelli and Scheffler et al. have carried out a series of studies to explicitly identify descriptors with the lowest possible dimensions for casual learning of material properties. Specifically, they firstly attempted to use the least absolute shrinkage and selection operator (LASSO) for feature selection.^[^ ^22f^ ^]^ LASSO constructs a regularization penalty function and recasts the complex problem into a convex minimization problem to provide a sparse solution for a large number of candidate features. Later, they employed the subgroup discovery (SGD) to find interpretable local patterns, correlations and descriptors of target properties.^[^ ^131^ ^]^ The process of SGD consists of three main steps: i) the use of a description language for identifying subgroups within a given data pool, ii) the definition of utility functions that formalize the quality of subgroups, and iii) the design of a Monte Carlo search algorithm to find selectors that describe the subgroups of interest. However, the first application of these CS‐based early numerical fitting methods is not for the heterogeneous catalysis with complex interface mechanism, but for the relatively simple bulk structures. Both mentioned works of LASSO and SGD classifies the crystal structures of the octet binary semiconductors into rock‐salt or zinc‐blende, which is a classic physically meaningful model. As a further development, SGD was applied to the neutral gas‐phase gold clusters to identify the general pattern between the geometrical and physicochemical properties. Nonetheless, they pointed out that in addition to the showcase applications demonstrated in the papers, LASSO is unable to handle the larger feature space of more than thousands of candidates. Simultaneously, they further proposed an improved systematic approach for discovering descriptors, i.e., sure independence screening and sparsifying operator (SISSO).^[^ ^88^ ^]^ This methodology was benchmarked with the quantitative prediction of the ground‐state enthalpies of octet binary semiconductors and applied to the showcase example of the metal/insulator classification of binaries. SISSO applies algebraic/functional operators such as addition, multiplication, exponentials, powers, roots, etc. to construct candidate features based on the basic physical quantities, and then recognizes the optimal effective descriptors from the feature space. Within the framework of CS‐based dimensionality reduction, SISSO can tackle immense feature space of billions or more and converge to the optimal solution of correlated feature combinations. The outcome of SISSO is a mathematical model in the form of an explicit analytical function of the input physical quantities, which improves the interpretability and generalizability of the model. Recently, they further made use of SISSO to identify several optimal descriptors for the adsorption energies of a series of adsorbates, including H*, C*, CH*, CO*, O*, and OH*, at all the potential surface sites of TMs, SAAs and bimetallic alloys.^[^ ^22j^ ^]^ Four classes of primary features corresponding to atom, bulk, surface and adsorption site were included for the feature construction in this work. Then they constructed the arbitrarily large feature spaces by iteratively applying these operators to the readily generated features, and the adjusted feature space is about 10^11^ after the third iteration Φ_3_, as shown in **Figure** **11**. Through the utilization of the sparsifying constraint, SISSO can identify the few best features out of the immense feature space. The size of the smaller feature subspace is equal to a user‐defined sure independence screening (SIS) value times the dimension of the descriptors. They found that when the size and complexity of feature space or the dimensionality of descriptors increases, the SISSO training errors systematically decrease and level out around the 5d to 8d descriptors. The suggested 8D, Φ_1_ descriptor has a good compromise of accuracy and complexity, with the observed validation RMSE of 0.18 eV compared to 0.15 eV of the 8D, Φ_3_ descriptor. This suggests that the appropriate descriptor can be transferred across both alloys and active site motifs without so complex mathematical form. This high‐throughput screening scheme also provides the energetics for the most stable and all metastable sites of substrates, and takes into account the kinetic barriers for a given catalytic reaction. In addition, the multi‐task learning used in this scheme can simultaneously identify common descriptors of adsorption energies of several different adsorbates, with a better prediction performance compared to the identification of separate descriptors for each adsorbate.^[^ ^132^ ^]^ It is worth noting that the characteristic parameter of adsorbates *α* in our intrinsic model has been successfully applied to describe various adsorbates on an equal footing in a single learning task. By introducing *α* as an input feature for SISSO, one may not only save the computational cost of multi‐task learning, but also analyze and explore the interplay between substrates and adsorbates.

Compared with other established methods (e.g., LASSO), SISSO shows superior advantages, especially when dealing with the immense highly correlated feature spaces. Currently, the problem of SISSO is the insufficient computer memory for the function space processing, and thus the more efficient implementation is required. Although other numerical fitting methods for constructing and identifying descriptors have not been widely used in heterogeneous catalysis, their excellent performance and potential are also worth the further exploration and application. However, despite their higher efficiency, the inherent difficulty for all the numerical fitting descriptors lies in the construction of the underlying physical picture. Compared with the existing physical picture‐based descriptors, numerical fitting descriptors have worse interpretability for the real adsorption mechanism of various alloy systems because of their methodological characteristics.

## 4 Discussion and Remarks

With the recent development of ML techniques, ML algorithms are helpful for the design of alloy catalysts, providing more significant opportunities in accelerating alloy catalysts discovery. Most importantly, the purpose of ML is to help one extract the physical picture from the immense data of heterogeneous catalysis to achieve accurate prediction. Although the prediction accuracy is gradually improved with the progress of research, the physical picture constructed by ML schemes is often inconsistent with the existing physical picture. This inconsistency is the critical issue for further improvement of accuracy and efficiency of ML applications, which demands more efforts on rational selection and further design of descriptors of alloy catalysts.

Heterogeneous catalysis is generally considered as a multi‐variable, multi‐scale and multi‐dimensional research issue, and the understanding of complex adsorption mechanism is the most effective approach to obtain catalytic performance. Adsorption is generally accepted as a local phenomenon, whose properties are mutually determined by adsorbates and substrates. At the atomic scale, the adsorption of adsorbates occurs at the local sites (e.g., top, bridge, and hollow) on substrate surfaces, which is generally considered to be affected mainly by the nearest and second nearest neighbor atoms of the surrounding environment. This local adsorption environment consisting of a central site and its neighbor atoms is often referred to as an active center of substrates.^[^ ^121^ ^]^ At the electronic scale, however, the variation of coordination environment originated from the overall alloying effect certainly affect the properties of the active center. What's more, the mechanisms of electron transfer in alloy catalysts and the electron exchange with adsorbates are probably non‐local and still controversial. In other words, although adsorption is known as a local phenomenon, its influencing factors are non‐local, especially for complex alloy substrates. Alloying effect is not a simple linear combination of intrinsic properties of different elements, and often leads to the unique catalytic performance of alloy surfaces, which is the key aspect to be concerned. Therefore, when the research systems extend from simple TMs to complex alloy surfaces, it is uncertain whether the originally effective descriptors will remain useful. The existing adsorption descriptors can be defined at different dimensions, depending on the specific problems in the research and the accuracy requirements of prediction. In this review, we put emphasis on the introduction of five types of alloy catalyst descriptors, which have been successfully used in ML schemes. For example, intrinsic BEPDs defined at the overall level cannot perceive and recognize the variations in the local environment of the adsorption sites of alloys. Due to the incapability of accurate capture of alloying effect, BEPDs are likely to fail to reveal the physical picture and realize accurate description of increasingly complex alloy systems. Therefore, descriptors are required to contain the corresponding information related to the local environment and be fine enough to encode the details of atomic level information. *d*‐band theory descriptors and local geometric descriptors go a step further in this regard, which make a more detailed description of electronic and geometric structures of alloy surfaces, respectively. The well‐known *d*‐band theory descriptors are originally designed for TM surfaces and do not perform well on alloys as known, and also can be used to describe the alloying effect with acceptable accuracy, owing to the theory refinement and the combination with ML techniques. Although the local geometric descriptors take into account the local environment variations, they ignore the changes of electronic structures caused by the alloying effect of different elements. These descriptors only describe a certain aspect of heterogeneous catalysis on the basis of their one‐sided perspectives, respectively. Our derived intrinsic descriptors, incorporating the electronic and geometric characteristics of both substrates and adsorbates, realize the more comprehensive description through the physical intuition of the adsorption mechanism. In our description, the local environment of active sites is averaged without considering the gradient of electronic and geometric structures. Although this averaging treatment can well describe the alloying effect of some alloys, it may not be accurate for the non‐local factors of adsorption for more complex alloy systems. CS based feature selection methods give an easier approach to find the simple numerical fitting descriptors. However, their relationship with catalyst properties and performance is still indirect and intricate, which is likely to yield a biased and unreliable description without utmost care. Although these descriptors have been successfully applied to various alloy systems in the corresponding papers, their validity still needs further verification when extended to other different complex alloy systems like HEAs and alloy NPs. This is probably mainly due to the unclear real physical picture of the controversial complex reaction mechanism on alloys. To reflect the physical picture and accurately predict catalytic performance simultaneously, it is crucial to generate or construct relevant chemical and physical features for a given problem. This situation demands the addition of more domain knowledge of heterogeneous catalysis. Further development of algorithms and techniques is also expected to accelerate the discovery of reactivity descriptors in the years to come.

## 5 Prospects

Some potential development directions for further ML research and rational design of descriptors for alloy catalysts are listed as below.

Intelligent feature selection and improvement of various descriptors: A descriptor is a low‐cost alternative model in the study of catalysts, which can be used to correlate some more complex figure of merit, such as the adsorption energy of key intermediates in heterogeneous catalysis. Organic incorporation of different types of descriptors should be considered to complement the description of more aspects of heterogeneous catalysis. More attention should be paid to the interactions among elements in alloys and their further connections and interactions with adsorbates. Taking our descriptor system for instance, the introduction of long‐range electronic and geometric effects, including their gradients, can be attempted to refine the original local averaging descriptors.^[^ ^129j^ ^]^ In addition, when our adsorbate parameter *α* is applied to the more complex molecules, its interactions with substrates need to be determined accordingly. For the well‐known *d*‐band theory descriptors, subsequent researches should consider a more comprehensive description of the *d*‐band shape to enrich the description of alloy surface properties. For simple local geometric descriptors such as CN and GCN, there have been many successful cases combining them together with electronic descriptors to capture catalytic properties of catalysts. Therefore, for those stronger local geometric descriptors, the organic combination with other electronic descriptors is particularly significant for the complement of description of electronic interactions among different elements. In short, an ideal description scheme should take into account influencing factors of all aspects, and reflect the real physical picture of heterogeneous catalysis. The complete form of ideal description should be relatively complex, while the corresponding simple form can be automatically derived when the considered system is determined. Moreover, the simple form should be able to be associated with the existing descriptor systems without conflicts. This ideal situation seems unlikely to happen, but with the increase of the richness of scenarios encountered, the new description schemes become more and more comprehensive. Refinement of the theory based on new observations can progressively improve the transferability, intrinsic capability and decision‐making ability of the cognitive system. The future development of ML tools for descriptor identification also can be expected to go beyond human intuition. In a word, although the discovery of a universal reactivity descriptor for all materials and reactions is not achievable in the short term, the existing descriptors and their further improvement are essential for the ultimate realization of the globally universal descriptor.

Material databases expansion and data share: ML indeed has the powerful prediction ability, but its establishment depends on the sufficient training data. Although the existing databases contain a large number of useful material data, there are more data in the published papers have not been entered into databases and shared. Therefore, a more comprehensive and general material information standard should be established to realize data sharing among databases and to reduce obstacles in data acquisition. Especially for a large amount of DFT calculation results, if these data cannot be unified on an equal footing due to different employed functionals and related parameters, it would be disadvantageous for researchers to analyze the underlying physical picture and to design universal descriptors for more systems. Moreover, natural language processing (NLP) techniques, which mainly deals with the effective communication between human and computer with human language, can be used to explore more useful information in a large number of published papers and to expand the existing databases.^[^ ^133^ ^]^

Rational selection and optimization of machine learning algorithms: According to the characteristics of the input data set, there are often some differences in the prediction results of different categories of ML algorithms. Therefore, to obtain the most appropriate algorithm and the real generalization error, one must take model selection as a part of the training process. Recent ML works have focused on the use of active learning in automated ML training.^[^ ^19^, ^89^, ^110^ ^]^ Active learning refers to the learning algorithm that can actively put forward some annotation requests and submit some filtered data to experts for annotation. In the case of catalyst design, experts’ annotation refers to the validation of DFT calculation results. Moreover, among diverse algorithms of ML and deep learning, numerous algorithms have been barely applied to heterogeneous catalysis, such as unsupervised learning,^[^ ^41^ ^]^ semi‐supervised learning,^[^ ^42^ ^]^ and reinforcement learning.^[^ ^43^ ^]^ When appropriate descriptors are constructed, these algorithms may also play an amazing performance in accurate prediction. On the other hand, the interpretability of ML algorithms themselves are also important and needs to be further improved. There are several methods to evaluate and analyze the role of features in ML schemes, for example, Pearson correlation coefficients are often used to analyze the correlation among features, and the decision tree‐based algorithms can evaluate the feature importance. However, these evaluations sometimes do not match the existing physical picture and are thus not convincing for the guide of heterogeneous catalyst design. Generally speaking, nowadays heterogeneous catalysis is still complicated enough to promote the development of novel techniques, and there is still plenty of space to design new ML models and invent new ML methods to better deal with the existing issues.

## 6 Summary

Over the past decades, a variety of ML methods have been developed, demonstrating their great advantage in solving the outstanding challenges of heterogeneous catalysis, which are difficult to solve by the conventional methods. In this review, we briefly retrospect the applications of ML methods in various alloy catalyst systems, and summarize several representative categories of descriptors. We have discussed the advantages and disadvantages of various ML algorithms and reactivity descriptors, which is crucial for the application of data‐driven methods in heterogeneous catalysis. A variety of successful examples have demonstrated their huge potential in achieving prediction accuracy, discovering new materials, and revealing structure‐property relationship based on the known material data. Through the various alloy systems and complex catalytic reactions, we not only clarify the existing understanding of the physical picture in the study of heterogeneous catalysis, but also emphasize the significance of rational selection of descriptors. Finally, we highlight the related challenges in the design of ideal universal descriptors and put forward the future development possibilities of descriptors, data and algorithms for the improvement of ML prediction schemes of alloy catalytic properties. It is our hope that this review helps readers to better understand the significance of ML scheme and descriptor design for the further researches of alloy catalysts.

## Conflict of Interest

The authors declare no conflict of interest.
